# COVID-19 pandemic and mental health care of older adults in India

**DOI:** 10.1017/S1041610220001441

**Published:** 2020-07-08

**Authors:** Vihang N. Vahia, Ashutosh B. Shah

**Affiliations:** 1 Dr. R. N. Cooper Hospital and HHT Medical College, Mumbai, India; 2 Sir H.N. Reliance Foundation Hospital, Mumbai, India

On March 11, 2020, the World Health Organization declared a pandemic status for the coronavirus disease 2019 (COVID-19) viral infection. India with a population of 1.3 billion people had reported its first case on January 30, 2020. India is a densely populated country. Government of India took the decision to promulgate countrywide lockdown since March 25, 2020, currently for a period of 3 weeks until April 14, 2020, thus curtailing social contacts and mobility. In this context, the older adults are confronted with challenges like non-availability of attendants to help them with tasks of daily living, difficulty in accessing medical help, and perceived apprehension of adverse course of existing morbidity. Repeated caution, publicized through the media and every other channel of communication of high risk of potentially fatal complications if infected with COVID-19 virus, increased sense of helplessness and anxiety. Those elderly who live within the traditional joint family system could avail of support of other family members in dealing with the emerging exigencies. In an average Indian household, four to five people live under one roof. Many families would have one or more persons above 60 years of age, one below 18 and two others aged somewhere in between. Three generations often live together and 75% of the Indian households (900 million) live in two rooms or fewer (Biswas, 2020). Hence, the risk of aerosol infection is a major public health risk. There are reports of instances where the neighbors quarantined the entire family to save the older adults (Vahia, 2020). However, there is a progressively increasing number of the older adults who live alone. The children may have migrated to another city or gone over to another country. These older adults depend upon hired house-help for their daily living and their neighborhood primary care physicians for health care. The extended family support activates when needed. After the start of lockdown, the older adults have to fend for themselves. In many instances, the domestic helper was not able to come over and the supply of cooked meals was not as easy. Media reported that during the first week of the 3-week lockdown, the number of people seeking help for mental health issues increased by 20% (Lolwal, 2020), the precise demographic breakup of the new help seekers is not available yet. Many people in India have stigma about mental illness and many prefer to seek alternative medicine remedies. Mental health awareness campaigns by multiple national and international celebrities, who publicly announced their mental illness and benefits of psychotropic medicines, has helped social acceptance of mental health care in India. The current COVID-19 virus pandemic has generated demand for psychiatric help. India has ~8000 mental health professionals most of them in urban areas. The older adults do not have an easy access to the mental health professionals. They are vulnerable to feeling lonely and helpless. They fear that even if they survive the pandemic, social order would change. Some fear worsening of morbidity and deterioration in quality of life.

The mortality reported so far from the current pandemic is less than that during the Spanish Flu of 1918, where 17 to 18 million Indians succumbed to the illness. However, economic recession has set in after the current pandemic. Mass unemployment and/or reduced wages will impair quality of life of the families and sense of insecurity prevails. Traditionally, the urban metros used to provide an economic security. COVID-19 pandemic has negated that security. Those who reverse-migrated to their native towns are likely to avail of support of the family and neighbors. The older adults who had to stay back in the metros are required to deal with loneliness, anxiety, and depression. As of now, we do not have the numbers of those who have migrated or precise strategies activated by the older adults to mitigate feelings of loneliness anxiety and depression.

Government of India and regional state governments are aware of the dilemma of the elderly. Several administrative steps to facilitate supplies, fiscal assistance, Internet-banking facility, and meal supplies may address their physical needs. Neighbors and NGOs try to organize help for the older residents. Media report of a Mumbai policeman helping a single octogenarian female suffering from diabetes mellitus by fetching not only her medications from pharmacy but also vegetables and cash from her bank are heartening to read (TNN, 2020). McKinsey Digital India report released in March 2019 revealed that India has 560 million Internet subscribers in 2018 (McKinsey Global Institute, 2019). A media report published on January 30 2020 reveals that India crossed the 500 million mark in end 2019 for number of smartphones users (News18, 2020). While the demographic data of the users are not available, it is widely known that the urban elderly widely use smartphones and specifically WhatsApp and Facebook to correspond with their family, friends, religious groups, neighborhood and to keep themselves abreast of local and global news. Smart phones are proving to be an effective channel of communication. At the same time, they are also a bane due to extensive sharing of misinformation. Several TV channels announce caution against misinformation that appears repeatedly on the social media. The Government agencies and news channels periodically refute the misinformation noticed on the social media and the cybercrime agencies take punitive actions. There is a continuous effort to minimize the angst among the elderly who remain vulnerable. Few local social and political leaders have organized virtual communication portals on WhatsApp to keep the residents including older adults well informed and to troubleshoot their expressed issues. Multiple clusters of mental health professionals have set up helplines all over the country. They collaborate with non-government organizations to set up these helplines and provide free mental health counseling via WhatsApp or phone. Several NGOs including Mission Zero COVID-2020 (Vahia, 2020), national and regional branches of Indian Psychiatric Society, Bombay Psychiatric Society, National Institute of Mental Health And Neurosciences (NIMHANS), Central Institute of Psychiatry (CIP), and Lokopriya Gopinath Bordoloi Regional Institute of Mental Health (LGBRIMH) (Vahia, 2020) have volunteered 24 × 7 helplines. The Board of administrators of the Medical Council of India, the apex medical regulatory body in India, has released telemedicine guidelines to facilitate the practice of telemedicine service for existing patients as well as new patients. The local medical licensing and governing bodies released circulars that legalized drug prescriptions during the lockdown period of medicines via email, WhatsApp, and other internet apps. Several corporate hospitals released mobile apps to facilitate virtual online consultations (Vahia, 2020).

A senior mental health counselor has shared three case vignettes where virtual communication and supportive therapy was effective among the older adults in her practice. One 72-year-old male, known case of hypertension and depression, was advised COVID-19 virus testing for symptoms of cough and fever. He tested positive and advised home quarantine. The patient stays with his spouse who tested negative. Their house help had stopped visiting due to lockdown and both their children live overseas. The middle socioeconomic group family had been seeking information about the COVID-19 from WhatsApp and the news channels. They said they “addicted” to surfing the social media though it compounded their anxiety, loneliness, and stress. The counselor was contacted to learn skills to deal with surge in symptoms of anxiety. The counselor held a video consultation to remedy their data bank of misinformation and help them to ventilate their thoughts, feelings, and apprehension partially worsened by the misinformation, fake news, and false alarms. She suggested schedules of daily chores and helped them establish phone contact with their sons and relatives. He reported significant improvement 5 days later. Counselor continues her sessions of supportive therapy for him and his wife. Her second case is a 62-year-old woman from upper socioeconomic strata, a known case of obsessive-compulsive disorder. Her obsessive, compulsive, and anxiety symptoms worsened when she was home quarantined after testing positive for COVID-19 virus infection following high fever. A schedule of video cognitive behavior therapy – exposure response prevention – was commenced, which promptly reduced the intensity of compulsive behavior. The third patient, a 65-year-old male from middle socioeconomic family, with no prior history of mental illness, had been hospital quarantined after testing positive for COVID-19 virus following bout of sneezing and fever. The patient experienced symptoms of panic, anxiety. He blamed everyone around him for infecting him. The counselor offered video consultation to the patient and managed to get permission from the treating consultant to allow for video calls by the patient’s family to the patient. These measures helped instill hope confidence and faith in the patient that he will get well. His mood stabilized and he is now getting ready for discharge.

One hopes that such online measures are here to stay and prove to be a boon especially for elderly who face mobility issues, those with healthcare access issue, and for the rural population. Online access for older adults, once harnessed, can help create online support groups, provide video counseling, and help design diagnostic and therapeutic services. The COVID-19 pandemic could thus prove to be a blessing in disguise for accelerating healthcare resource and access issues faced by the population in India at large and the elderly in particular.
